# On-chip wavefront shaping in spacing-varied waveguide arrays

**DOI:** 10.1515/nanoph-2023-0323

**Published:** 2023-08-31

**Authors:** Yunfei Niu, Yunlong Niu, Xiaopeng Hu, Yong Hu, Qingyang Du, Shaoliang Yu, Tao Chu

**Affiliations:** Research Center for Intelligent Optoelectronic Computing, Zhejiang Laboratory, Hangzhou 311100, China; College of Control Science and Engineering, Zhejiang University, Hangzhou 310027, China; Radiation Monitoring Technical Center, Ministry of Ecology and Environment, Hangzhou, 310012, China; National Laboratory of Solid State Microstructures, and College of Engineering and Applied Sciences, Nanjing University, Nanjing 210093, China; College of Information Science and Electronic Engineering, Zhejiang University, Hangzhou 310027, China

**Keywords:** integrated photonics, wavefront shaping, waveguide array

## Abstract

The ability to manipulate light propagation sets the foundations for optical communication and information processing systems. With the ever-growing data capacity and data rate, photonic integrated circuits have attracted increasing attentions of researchers owing to their large-volume integration capacity and fast operation speed. In this work, we proposed and experimentally demonstrated a new wavefront shaping method using waveguide arrays with hyperbolic secant refractive index profiles. Through theoretically analyzing the diffraction and coherence properties, we found that a single waveguide array can perform both imaging and phase transformation, which are the two primary functions of optical lenses. We further expanded this function and fabricated the corresponding devices on a silicon nitride waveguide platform. Deterministic beam shaping, such as focusing, expansion, collimation, and steering, is successfully realized. This wavefront control method exhibits the potential for on-chip optical routing, ranging, sensing, etc., with high integration density and scalability.

## 1 Introduction

The utilization of wavefront shaping technology has proven to be a highly efficient approach for manipulating the propagation of light. This technology enables the customization of light amplitude, polarization, and phase [1–9]. Despite its demonstrated efficacy, the use of this technology in waveguide systems has been limited because of the difference in scale between the shaped wavefront and the waveguide dimensions [10]. The integration of this technology into waveguide platforms, which are considered the fundamental building blocks of integrated photonics, could offer new opportunities for precisely shaping optical signals in photonic circuits. To achieve on-chip wavefront shaping, it is necessary to simultaneously manage the diffraction and also modulate the phase [11]. On-chip diffraction manipulation has been comprehensively explored through kinds of waveguide systems, such as dielectric waveguides with a height gradient [12], curved waveguide arrays [13], plasmonic waveguides [14, 15], and photonic crystal waveguides [16]. Meanwhile, a precise phase compensation design is typically accomplished through gradient or graded index (GRIN) structures, such as sub-wavelength GRIN waveguide arrays [17, 18], continuous GRIN lenses [8, 19, 20], and gradient metasurfaces [21–23]. It is worth noting that, all those works only achieved either one function, it still remains challenging to achieve precise management of both the effective index profile and the lateral confinement simultaneously in waveguide systems.

Arrayed waveguides stand out as a promising solution for on-chip wavefront shaping. Waveguide arrays have proven to be highly effective for a range of on-chip applications, including imaging [13, 24, 25], light detection and ranging (LiDAR) [11, 26], [27], [28], quantum system simulation [29–34], and multiport information processing [35–38]. In wavefront shaping, compensating for the phase of the wavefront and confining the wavevector laterally (perpendicular to the waveguide direction) in arrayed waveguide systems is a challenging task due to the inherent diffraction nature of light. This work demonstrates the successful implementation of on-chip wavefront shaping through the use of dielectric spacing-varied waveguide arrays. Our preliminary investigation into the diffraction properties of these arrays has revealed the potential to manipulate the lateral wave vector as light propagates. Prior techniques for diffraction management in similar optical systems have relied on adjusting the effective coupling coefficient [13, 16] or altering the angle of incidence of light [39, 40]. In contrast, we have developed a new method for managing on-chip diffraction by utilizing waveguide arrays with hyperbolic secant index profiles. This approach enables the control of the lateral wave vector by introducing normal and anomalous diffraction at various transverse positions, thereby allowing for exact focusing, expansion, collimation, and steering of the light beam. Furthermore, spacing-varied waveguide arrays are well suited for phase transformation, enabling both phase modulation and imaging with a single array. Our experiments have confirmed the equivalence between the proposed spacing-varied waveguide array and lens. This was observed through the phase modulation effect of the input light with different phase profiles. Our approach is scalable and can be applied to high-density integration, particularly in applications that necessitate multiple inputs/outputs, such as integrated photonic neural networks [41–43] and on-chip switch networks [44]. The phase modulation effect of the spacing-varied waveguide array also holds potential for more complex on-chip information systems, such as on-chip 4-f systems [17, 45, 46].

## 2 Theory

**Figure 1:**
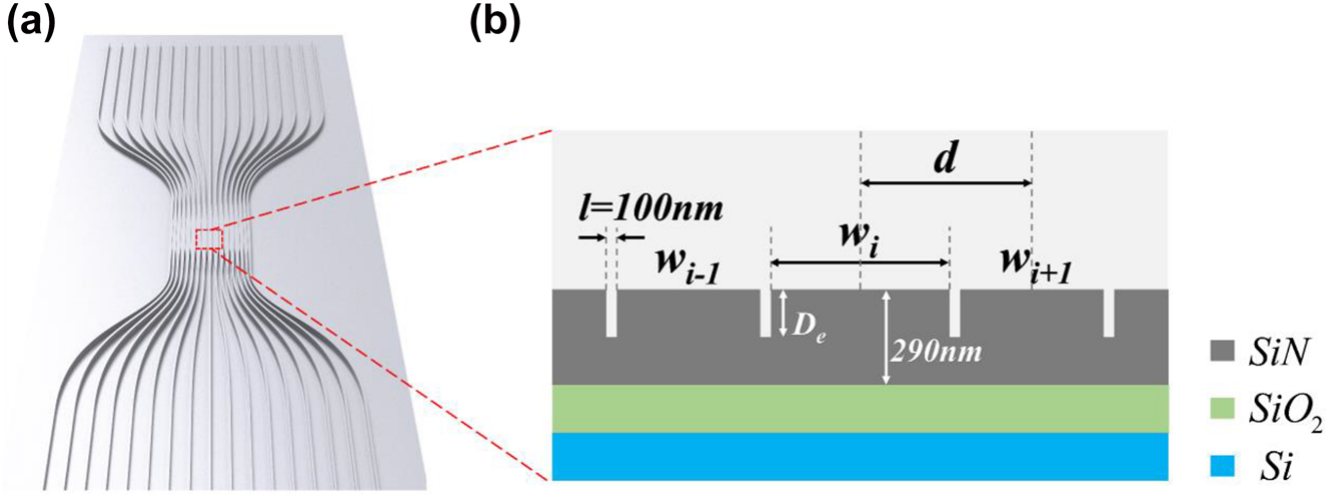
Schematic illustration of the dielectric spacing-varied waveguide array. (a) A scheme of the waveguide array structure. (b) Corresponding cross-sectional view at the coupling region.

The diffraction characteristics of specific optical systems have been extensively studied [39, 47, 48]. In this work, a spacing-varied waveguide array with a gradient refractive index profile was utilized to realize on-chip wavefront shaping. A schematic drawing illustrating such a structure is presented in Figure 1(a). The array consists of 17 waveguides that were connected to independent fan-in and fan-out optical ports. A cross-sectional view of the waveguides is depicted in Figure 1(b) with a 290 nm thick silicon nitride thin film platform. In our design, the gap between each of the two waveguides was set to be 100 nm. The etching depth of the waveguide array, *D*_e_, plays a crucial role in managing diffraction. The waveguide width distribution symmetrically decreases from the center and adheres to a quasi-hyperbolic secant profile.

The initial analysis focused on the diffraction characteristics of the spacing-varied waveguide array. Based on optical coupled-mode theory, the equations that describe the electric field in the *n*th waveguide and the diffraction relation are expressed as [39]:

(1) $$\frac{dE_{n}}{dz}=ik_{wg}E_{n}+iC\left(E_{n-1}+E_{n+1}\right),$$

(2) $$k_{z}=k_{\text{wg}}+2C\cos\left(k_{x}d\right).$$

where *E*_*n*_ is the electric field in the *n*th waveguide, *k*_wg_ is the propagation constant, *C* is the coupling constant between adjacent waveguides, *k*_*z*_ and *k*_*x*_ are wave vectors along and perpendicular to the waveguide direction, respectively, and *d* is the distance between the adjacent waveguide centers. Next, the diffraction of the waveguide array is defined as [39]:

(3) $$D=\frac{1}{z}\frac{\partial^{2}\phi }{\partial k_{x}^{2}}=\frac{\partial^{2}k_{z}}{\partial k_{x}^{2}}=-2Cd^{2}\cos\left(k_{x}d\right)$$

**Figure 2:**
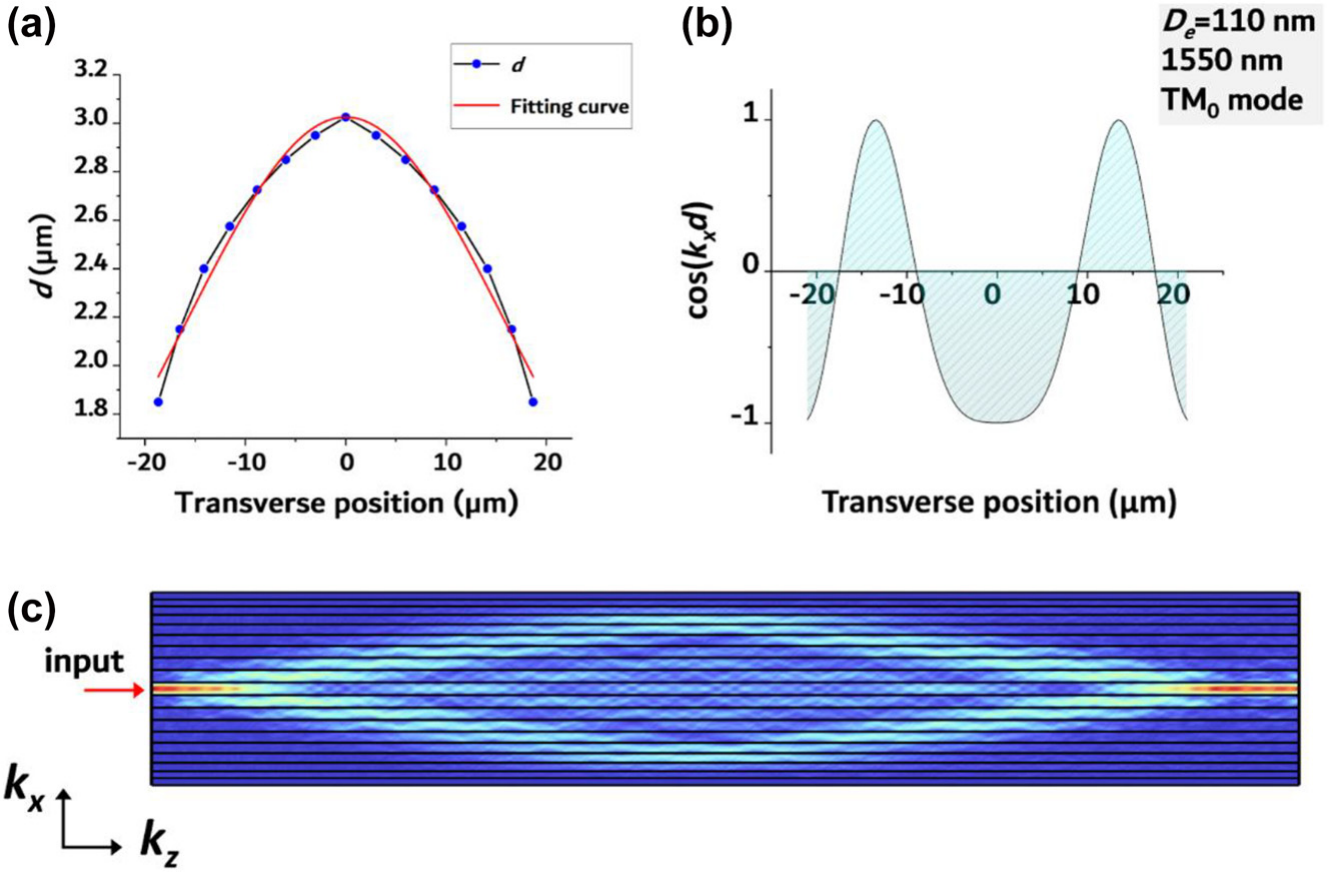
Schematic illustration of the dielectric spacing-varied waveguide array. (a) Profile of “*d*” along the transverse direction, with a fitting curve that utilizes the hyperbolic secant function. (b) Result of calculated cos(*k*_*x*_ *d*). The curve corresponds to a 290 nm thick SiN waveguide array with an etching depth of 110 nm. The wavelength is 1550 nm, and the propagation mode is TM_0_. (c) Simulation result of TM_0_ mode propagation in the waveguide array. The electric field refocuses after a certain distance *L*.

It worth highlighting that while diffraction in free space is typically negative (dispersing), a unique feature of discrete diffraction is that it allows positive value and referred to as anomalous diffraction, which concentrating beams during diffraction [39]. In general, there have been two methods developed for diffraction management in the past few decades. One involves changing the sign of *C*, as is demonstrated in photonic crystals [16, 49] and sub-wavelength waveguide arrays [13, 14]. The other modulates *k*_*x*_, which can be accomplished in uniformly curved waveguide arrays [50]. We, instead, proposed a new approach to discrete diffraction management leveraging spacing-varied waveguide arrays. The width distribution in the spacing-varied waveguide array has a shape similar to that of a hyperbolic secant, resulting in a quasi-hyperbolic secant profile for *d*(*x*) as is seen in Figure 2(a). The discrete diffraction property of the spacing-varied waveguide array is determined by the sign of cos(*k*_*x*_ *d*). We used COMSOL Multiphysics to simulate *k*_*x*_ and calculated cos(*k*_*x*_ *d*) for the spacing-varied waveguide array with an etching depth of 110 nm and a working wavelength of 1550 nm for the TM_0_ mode. The results are displayed in Figure 2(b). It is evident that such spacing-varied waveguide array clearly generated opposing signs for *D*(*x*). Designing uneven distribution of cos(*k*_*x*_ *d*) is the main approach for diffraction management. The focusing effect is a direct consequence of the cos(*k*_*x*_ *d*) profile shown in Figure 2(b). Simulation result of its propagation properties is presented in Figure 2(c). TM_0_ mode at 1550 nm was injected at the center waveguide’s input port. The light spreads out but then refocuses back into the initial waveguide after traveling a distance of *L*. This focusing effect can be explained by the fact that at the beginning of propagation, diffraction is abnormal and causes the wavefront to expand. However, once the wavefront enters the normal diffraction area, the normal diffraction begins to counteract the abnormal diffraction, ultimately resulting in a focusing effect. It’s worth noting that once *d*(*x*) is established, the only factor that affects the cos(*k*_*x*_ *d*) profile is *k*_*x*_, which changes with wavelength and waveguide mode. As a result, when the input light wavelength or propagation mode is altered within the same spacing-varied waveguide array, different diffraction patterns are expected.

**Figure 3:**
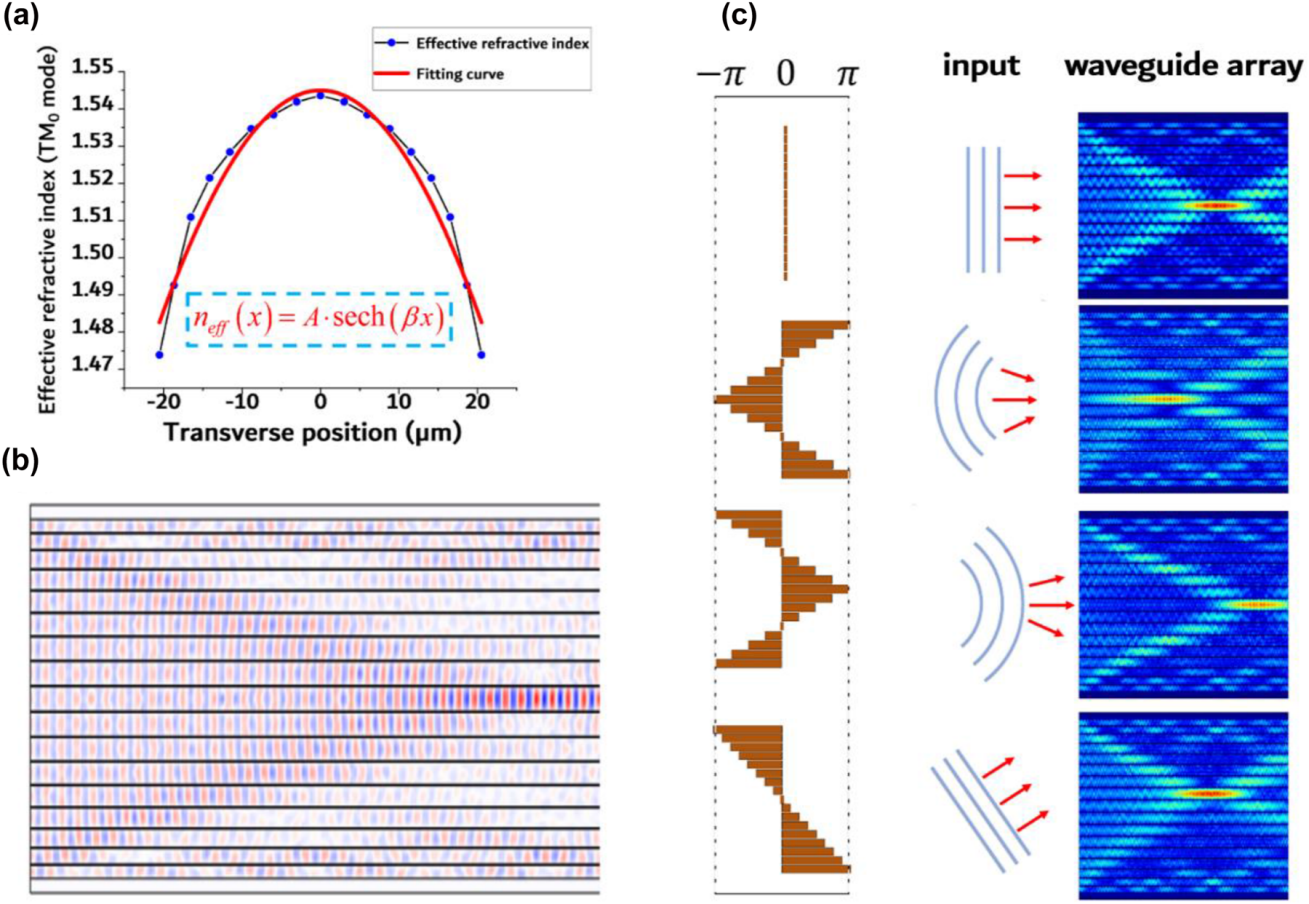
Phase modulation in spacing-varied waveguide arrays. (a) The effective refractive index profile of the 1550 nm TM_0_ mode. (b) Electric field distribution for planar light input into spacing-varied waveguide array. The mode considered is TM_0_ at 1550 nm. (c) Simulation result of light propagation in spacing-varied waveguide arrays with varying input phase profiles.

The spacing-varied waveguide array has a noteworthy phase modulation effect. Its effective refractive index profile of the 1550 nm TM_0_ mode is depicted in Figure 3(a). The effective refractive index function can be approximated by the hyperbolic secant curve and its first-order approximation of the Taylor expansion series:

(4) $$n_{\text{eff}}\left(x\right)=A\cdot sech\left(\beta x\right)\approx A\cdot \left(1-\frac{{\left(\beta x\right)}^{2}}{2}\right)$$

Thus, the spacing-varied waveguide array has an additional phase accumulation of:

(5) $$\varphi_{\text{WA}}=\exp\left(i\frac{2\pi }{\lambda }n_{\text{eff}}\left(x\right)\cdot z\right)\approx \exp\left(A\cdot \left(\left(i\frac{2\pi }{\lambda }\cdot z\right)-i\frac{2\pi }{\lambda }\frac{{\left(\beta x\right)}^{2}}{2}\cdot z\right)\right)$$

The parabolic distributed *φ*_WA_ has the same form as that of thin lenses in space, which is [51]:

(6) $$\varphi_{\text{lenses}}=\exp\left(-i\frac{2\pi }{\lambda }\cdot \left(\frac{x^{2}}{2f}\right)\cdot z\right)$$

Therefore, spacing-varied waveguide shares the same focusing effect as a free space lens. We conducted a simulation of light propagation in spacing-varied waveguide arrays using a plane wave input, and the results are displayed in Figure 3(b). By modifying the phase profile of the input plane wave, various electric field distributions can be achieved, as demonstrated in Figure 3(c). The phase profiles were adjusted to imitate the plane wave, focusing wave, diverging wave, and oblique incident wave, respectively. The simulation results clearly indicate that the spacing-varied waveguide arrays function as free-space lenses in both phase modulation and imaging.

## 3 Experimental results

**Figure 4:**
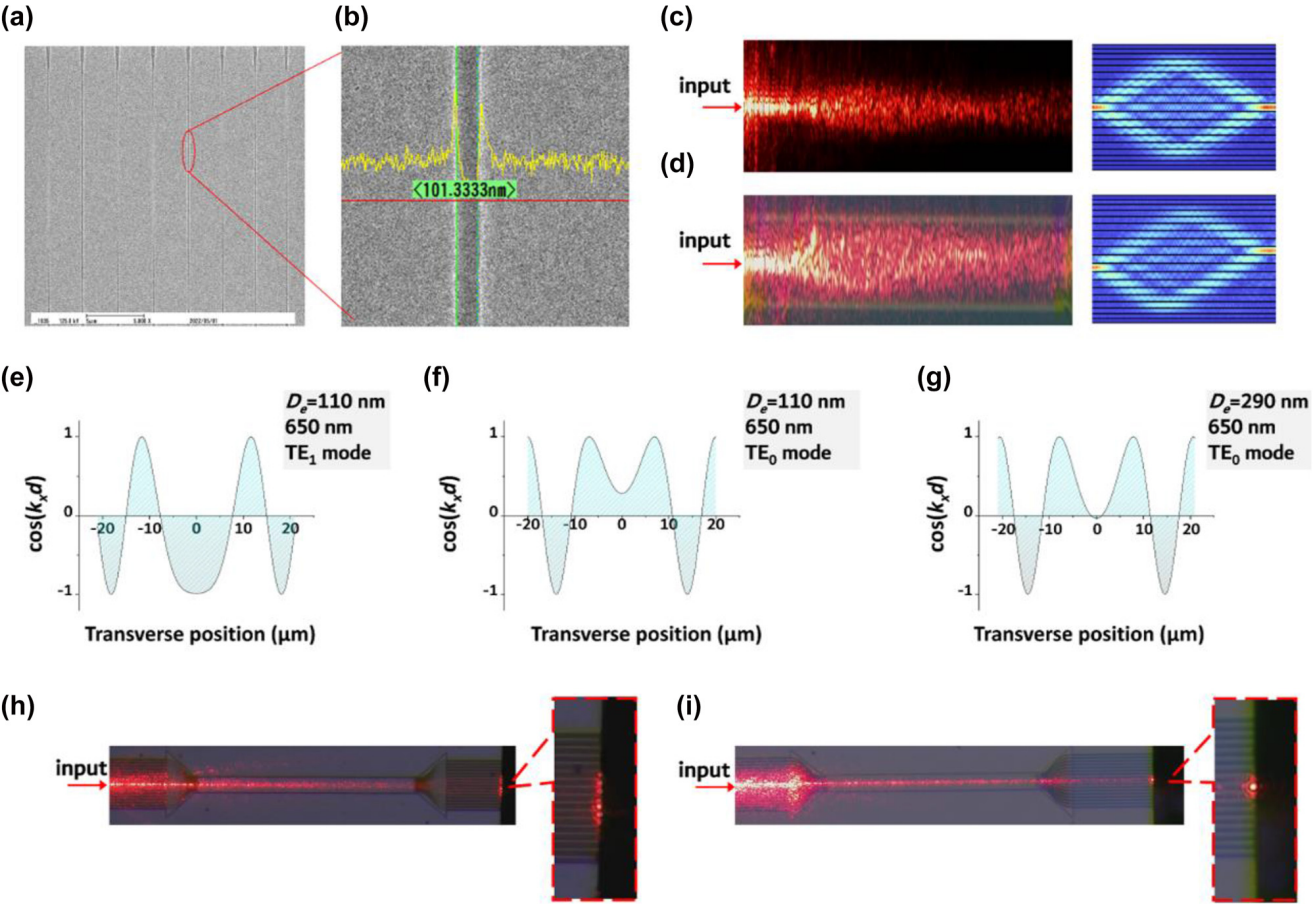
Experiment results. (a) and (b) The SEM characterization results of the fabricated SiN spacing-varied waveguide arrays. The gap between adjacent waveguides was about 100 nm. SEM pictures show the fine quality of the samples. (c) Surface scattering of the SiN spacing-varied waveguide array with 650 nm light input from the intermediate waveguide. Its simulation correspondence is on the right. The propagation mode was TE_1_. (d) Surface scattering of the SiN spacing-varied waveguide array with 650 nm light input from the waveguide right next to the intermediate waveguide. Its simulation correspondence is on the right. The propagation mode was TE_1_. (e) cos(*k*_*x*_ *d*) profile of the SiN spacing-varied waveguide arrays with an etching depth *D*_e_ = 110 nm at 650 nm, the propagation mode was TE_1_. (f) and (g) cos(*k*_*x*_ *d*) profile of the SiN spacing-varied waveguide arrays at 650 nm. The propagation mode was TE_0_, and the etching depths *D*_e_ in (f) and (g) were 110 nm and 290 nm, respectively. (h) and (i) Surface scattering of 650 nm red light propagation in the SiN spacing-varied waveguide array. The etching depth *D*_e_ was 110 nm and 290 nm in (h) and (i), respectively. The propagation mode was TE_0_ in both cases.

In order to validate the simulated effects of diffraction management and phase modulation, we fabricated a set of silicon nitride spacing-varied waveguide arrays. The process involved depositing a 2 μm buried oxide (BOX) buffer on Si wafers, followed by a 290 nm thick SiN thin film deposition using plasma-enhanced chemical vapor deposition (Oxford Instruments, Plasmalab system 80 plus). It is worth noting that the refractive index of the silicon nitride thin films was measured using a spectroscopic ellipsometer (Sentech, SER 850 DUV). The refractive index value utilized in the aforementioned simulation is obtained from the fitted dispersion curve. E-beam lithography (Elionix, ELS125-G8) and dry etching (ULVAC, CE-300I) were then used to create the waveguide array pattern. We directly employed an electron beam exposed photoresist (positive-E-Beam Resists AR-P 6200) as the etching mask, resulting in ideal etching results. SEM images of the fabricated waveguide arrays are shown in Figure 4(a) and (b). The distance between adjacent waveguides is approximately 100 nm as designed. The SiN spacing-varied waveguide arrays had smooth edges and were free from parasitic etching residue, as seen in Figure 4(b). The chip’s facets were then polished, and a lensed fiber was used to couple light in and out of the waveguide arrays. In our experiment, we implemented both red light (650 nm, TOPFORZA, FT-6101) and infrared light (1550 nm, SANTECH, TSL550) to illustrate the phase modulation effect. The visible nature of red light endows the ability for directly observing light intensity distribution during propagation, while the infrared light was used to measure the transmission property owing to its low scattering loss. We first investigated light distribution during transmission in waveguide arrays that have an etching depth of 110 nm. Figure 4(c) and (d) display the light field distribution among the spacing-varied waveguide arrays when the red light was introduced into the center waveguide and its neighboring waveguide, respectively. Their corresponding simulation results are presented on the right. The partial uncollimated light at the input facet caused scattering on the surface of the waveguide array, resulting in a blurred distribution of the scattered light field originating from the waveguide array itself. The spacing-varied waveguide array’s length is 700 μm, and its TE_1_ mode was excited by modifying the relative position between the lensed fiber and the waveguide facet. We specifically chose TE_1_ mode of the red light as it yields a *D*(*x*) of opposing sign similar to that in a 1550 nm TM_0_ mode. Despite the presence of negative values of cos(*k*_*x*_ *d*) near the edge for the TE_0_ mode, as is simulated in Figure 4(f), they do not have sufficient impact to fully counteract the positive diffraction. Thus, focusing is not anticipated in the 700 μm long array for TE_0_ mode. On the contrary, the focusing effect was clearly observed for TE_1_ mode as in Figure 4(c). Figure 4(e) displays its cos(*k*_*x*_ *d*) profile, which illustrates the source of the focusing phenomenon characterized by an uneven distribution across the transverse direction. The focusing effect is a consequence of the equilibrium between normal and anomalous diffraction. The theoretical calculation of the focusing length takes the following form [12]:

(7) $$L=\frac{\pi }{\beta }$$

where *β* is the fitting parameter in Eq. (4). The calculated *L* for the 650 nm TE_1_ mode is approximately 406 μm, but the experimental results show a focusing length range of 450–500 μm. The differences are attributed to two reasons: (1) the spacing-varied waveguide arrays’ cos(*k*_*x*_ *d*) profile deviates from a perfect hyperbolic secant function and (2) fabrication errors during lithography and dry etching processes. The latter is considered the dominant cause, as dry etching depth can deviate up to 20 nm based on characterization results. In fact, the depth of the etching has a significant impact on the cos(*k*_*x*_ *d*) profile of the spacing-varied waveguide array, making it a crucial factor in diffraction modulation. To illustrate, we conducted simulations of the cos(*k*_*x*_ *d*) profiles of the 650 nm TE_0_ mode with varying etching depths, as depicted in Figure 4(f) and (g). It should be noted that when the etching depth is 110 nm, the 650 nm TE_0_ mode has a positive cos(*k*_*x*_ *d*) value that extends over 20 μm in the transverse direction. Despite the presence of negative values of cos(*k*_*x*_ *d*) near the edge, they do not have sufficient impact to fully counteract the positive diffraction. Thus, focusing is not anticipated in the 700 μm long array where the TE_1_ mode is focused. This is confirmed by experimental evidence in Figure 4(h), which shows an apparent expansion of the 650 nm TE_0_ mode in the array. The scenario changes in a waveguide array that has been etched at 290 nm, as the cos(*k*_*x*_ *d*) profile of the TE_0_ mode at 650 nm has nodes around the center, suggesting the possibility of zero diffraction. When red light was introduced into the intermediate waveguide of this array, a nondiffraction phenomenon occurred with the generation of the TE_0_ mode, as depicted in Figure 4(i). The results shown in Figure 4(c), (d), (h) and (i) demonstrate the ability to manipulate lateral vector confinement in a flexible manner, allowing for precise focusing, expansion, and collimation by selecting different cos(*k*_*x*_ *d*) profiles.

**Figure 5:**
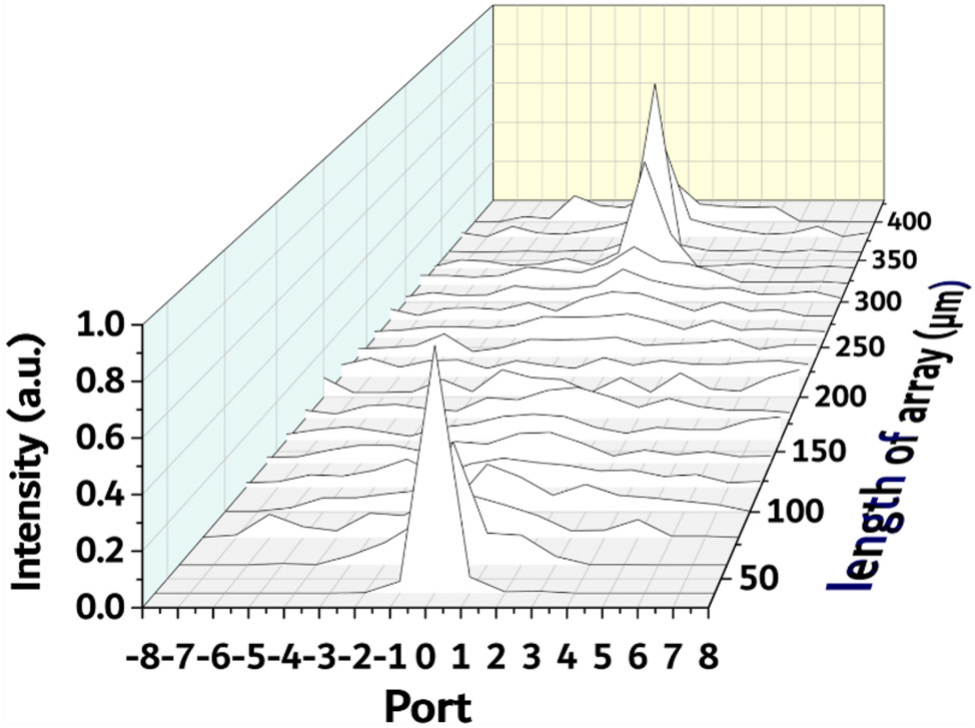
Normalized intensity profile of light propagation within the arrays at 1550 nm with TM_0_ mode. The length of the waveguide arrays ranges from 20 μm to 400 μm, with a length step of 20 μm, and an etching depth of 110 nm.

Next, we further investigated the transmission property of infrared light propagates in spacing-varied waveguide arrays. Figure 2(b) anticipated focusing to occur when the TM_0_ mode of 1550 nm propagates in a SiN spacing-varied waveguide array with an etching depth of 110 nm. We fabricated multiple spacing-varied waveguide arrays with varying waveguide lengths between 20 μm and 400 μm, with an increment of 20 μm. The polishing of the waveguide end faces was performed mechanically using various levels of fine-grit sandpaper. A fiber lens was employed for light collection, which was directly connected to a power meter for light intensity detection. The fiber lens (CXFIBER) used is a single-mode fiber designed for the 1550 nm wavelength with a focal spot size of 3 μm and focal length of 10 μm. By analyzing the power of the 17 output ports in each array, we generated a graph displaying the normalized intensity profile of light propagation within the arrays. The resulting normalized intensity profile, as seen in Figure 5, illustrates that the light initially diffracts and expands from the center waveguide at the beginning. However, after traveling a distance of approximately 180 μm, it starts to refocus. In the spacing-varied waveguide array, which is 360 μm long, the center waveguide collects around 82 % of the total output power.

**Figure 6:**
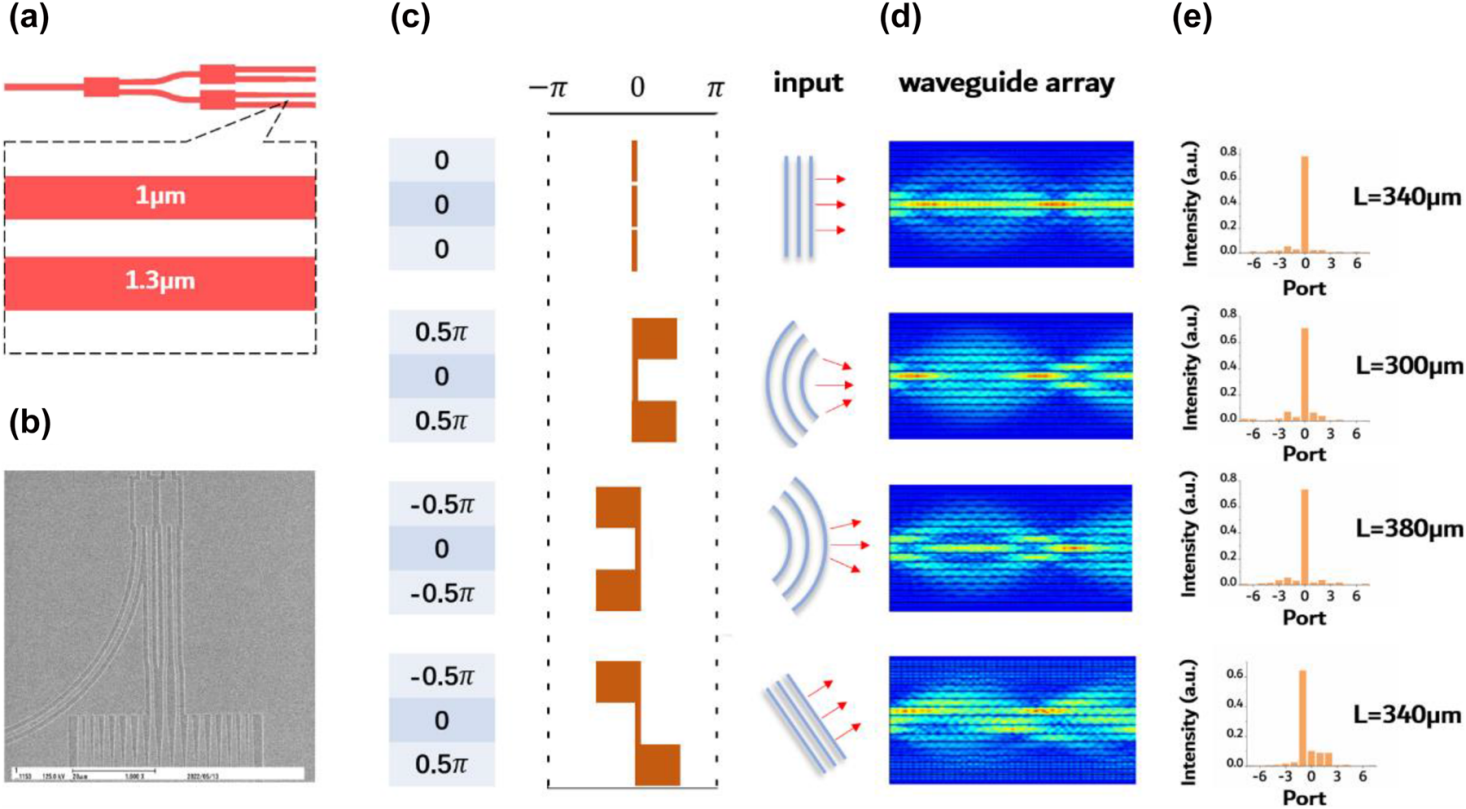
Design and experimental results of phase transformation in spacing-varied waveguide arrays. (a) Illustration of cascaded MMIs and phase shifters. The width of the phase shifter is 1 μm and 1.3 μm, respectively, with a length of 10 μm. (b) SEM of the fabricated MMIs and phase shifters. (c) Schematic illustration of the designed input phase plane. (d) Simulation results correspond to the four input phase planes. (e) Experimental results of the 1550 nm TM_0_ mode propagated in spacing-varied waveguide arrays, corresponding to the phase plane designed in (c).

Besides diffraction management, phase transformation was also investigated. We used cascaded multimode interferometers (MMI) to distribute light into three waveguides and manipulate the phase of the input light by adjusting the width of the input waveguides, as illustrated in Figure 6(a). The SEM images of the fabricated MMIs are shown in Figure 6(b). The input phase plane was designed to mimic plane waves, converging waves, diverging waves, and angled plane waves, as depicted in Figure 6(c). The effect of focusing and phase transformation in spacing-varied waveguide arrays on-chip is similar to that of a lens in free space, indicating that wavefront shaping on-chip is possible by adjusting input phase planes. Simulation results in Figure 6(d) show that plane, converging, and diverging waves will all focus on the center waveguide with different focal spot. Angled plane waves will focus on the upper waveguide with the same focal length as the plane wave. This phenomenon was further experimentally demonstrated in spacing-varied waveguide arrays of varying lengths, with a propagation mode of TM_0_ at 1550 nm. As shown in Figure 6(e), a focal length of 340 μm, 300 μm, and 380 μm for plane, converging, and diverging waves was observed, respectively. Same focal effect for the angled plane wave was also recorded as the majority of its power focused on the upper waveguide in the 340 μm long array.

## 4 Conclusions

In this work, we focused on the implementation of on-chip wavefront shaping in arrayed waveguide platforms. The diffraction and phase transformation properties of spacing-varied waveguide arrays were systematically investigated and successfully applied to wavefront shaping. A specific cos(*k*_*x*_ *d*) profile was utilized to manage diffraction, resulting in both normal and anomalous diffraction in a single array, and enabling lateral wavevector control. The study demonstrated precise beam focusing, expansion, and collimation in a series of SiN spacing-varied waveguide arrays, with a refocusing efficiency up to 82 % in a 360 μm long spacing-varied waveguide array. Additionally, spacing-varied waveguide arrays possess a gradient index profile that is advantageous for phase transformation. Simulations were conducted to observe light propagation in spacing-varied waveguide arrays with various input phase planes. By adopting cascaded MMIs and phase shifters, different input phase planes were created, and a beam steering function similar to that of a lens in free space was demonstrated. These findings suggest that spacing-varied waveguide arrays have the potential as candidates for on-chip wavefront shaping and can behave similarly to free-space lenses, providing new options for on-chip information processing. Furthermore, waveguide arrays are inherently scalable, making them highly promising for high-density integration, particularly for multiport applications.

## Supplementary Material

Supplementary Material Details
