# Supplementary material for: On-chip wavefront shaping in spacing-varied waveguide arrays
Source: Nanophotonics. 2023 Aug 31;12(19):3737–45. doi: 10.1515/nanoph-2023-0323 (PMC11636421; doi:10.1515/nanoph-2023-0323)
Supplement: Supplementary file 1 — Supplementary Material Details [file j_nanoph-2023-0323_suppl_001.docx]

**Supporting Information:** **On-chip wavefront shaping in spacing-varied waveguide arrays**

**Yunfei Niu^1,*^, Yunlong Niu^2,3^, Xiaopeng Hu^4^, Yong Hu^1^, Qingyang Du^1^, Shaoliang Yu^1^ and Tao Chu^5,*^**

^1^*Research Center for Intelligent Optoelectronic Computing, Zhejiang Laboratory, Hangzhou 311100, China*

^2^*College of Control Science and Engineering, Zhejiang University, Hangzhou 310027, China*

^3^Radiation Monitoring Technical Center, Ministry of Ecology and Environment, Hangzhou, 310012，China

^4^National Laboratory of Solid State Microstructures, and College of Engineering and Applied Sciences, Nanjing University, Nanjing 210093, China

^5^*College of Information Science and Electronic Engineering, Zhejiang University, Hangzhou 310027, China*

**Corresponding author: niuyunfei@zhejianglab.com,* [*chutao@zju.edu.cn*](mailto:chutao@zju.edu.cn)

CONTENTS

I. Dispersion characterization of the fabricated SiN thin film

II. Simulation results for spacing-varied waveguide arrays consists of 29 waveguides

III. Experiment result for red light transmission in outmost waveguide

IV. Characterization of the fabricated waveguide arrays

**I. Dispersion characterization of the fabricated SiN thin film**

In the process of fabricating our SiN thin film, we used plasma-enhanced chemical vapor deposition (PECVD) (Oxford Instruments, Plasmalab system 80 plus) to grow a 290 nm thick silicon nitride film on a silicon substrate. Refractive index measurements of the silicon nitride thin films were obtained using a spectroscopic ellipsometer (Sentech, SER 850 DUV). The fitted dispersion curve for the refractive index is shown in Fig. S1. Based on this curve, we set the refractive index values of SiN for wavelengths of 650 nm and 1550 nm at 2.0527 and 1.9944, respectively, for use in our simulations.


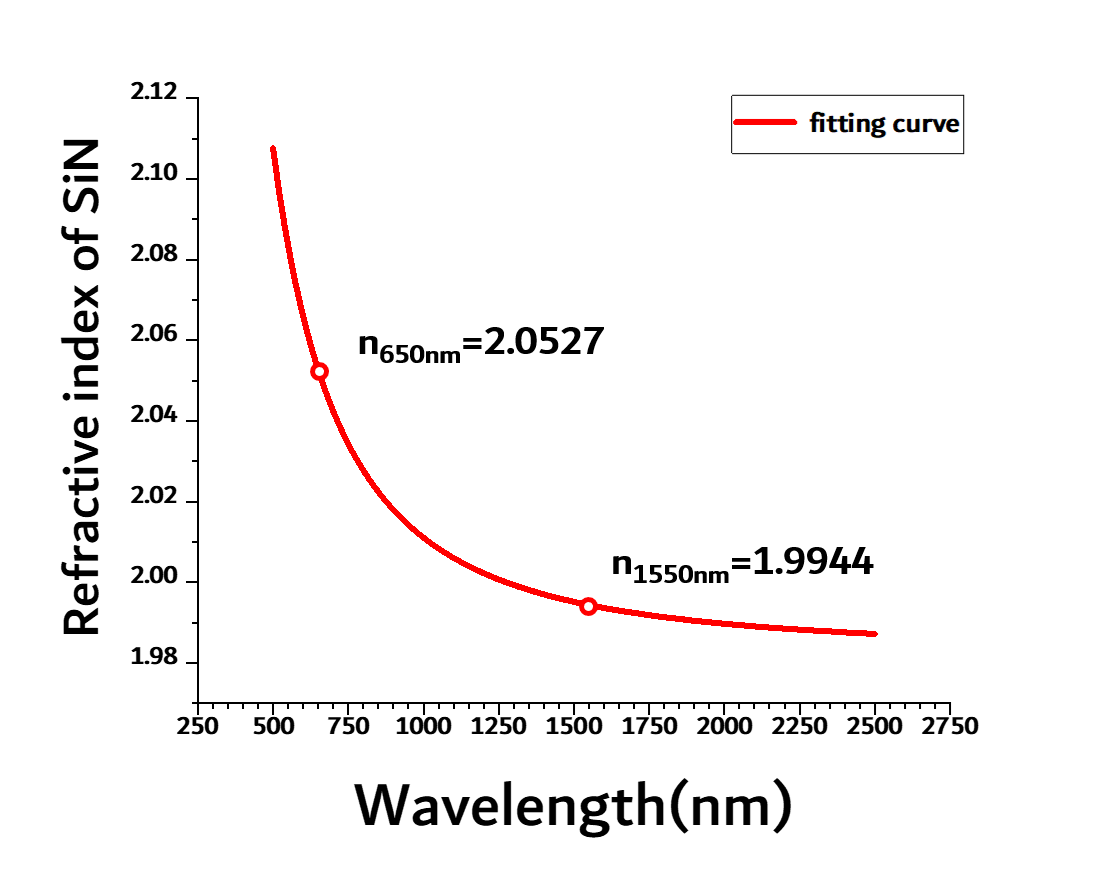
Figure S1: The fitted dispersion curve for the refractive index of the fabricated SiN thin film.

**II. Simulation results for spacing-varied waveguide array consists of 29 waveguides**

To demonstrate the advantages of the proposed spacing-varied waveguide array system for high-density, multi-port photonic integrated devices, we performed simulations on the light propagation in spacing-varied SiN waveguide arrays with 29 waveguides. The simulations were conducted at a wavelength of 1550 nm, considering the TM_0_ mode. The results of these simulations are shown in Fig. S2. In order to achieve wavefront shaping, we carefully selected the profile of d(x) and adjusted the waveguide width accordingly. The waveguide width profile is shown in Fig. S2(a), while the corresponding cos(k_x_d) profile is displayed in Fig. S2(b). Subsequently, we simulated the propagation of light in these spacing-varied waveguide arrays with varying input phase profiles, as depicted in Fig. S2(c). The phase profiles were adjusted to emulate plane waves, focusing waves, diverging waves, and oblique incident waves. The simulation results demonstrate that spacing-varied waveguide arrays composed of 29 waveguides can effectively achieve wavefront shaping, indicating the scalability and efficiency of the proposed design approach.


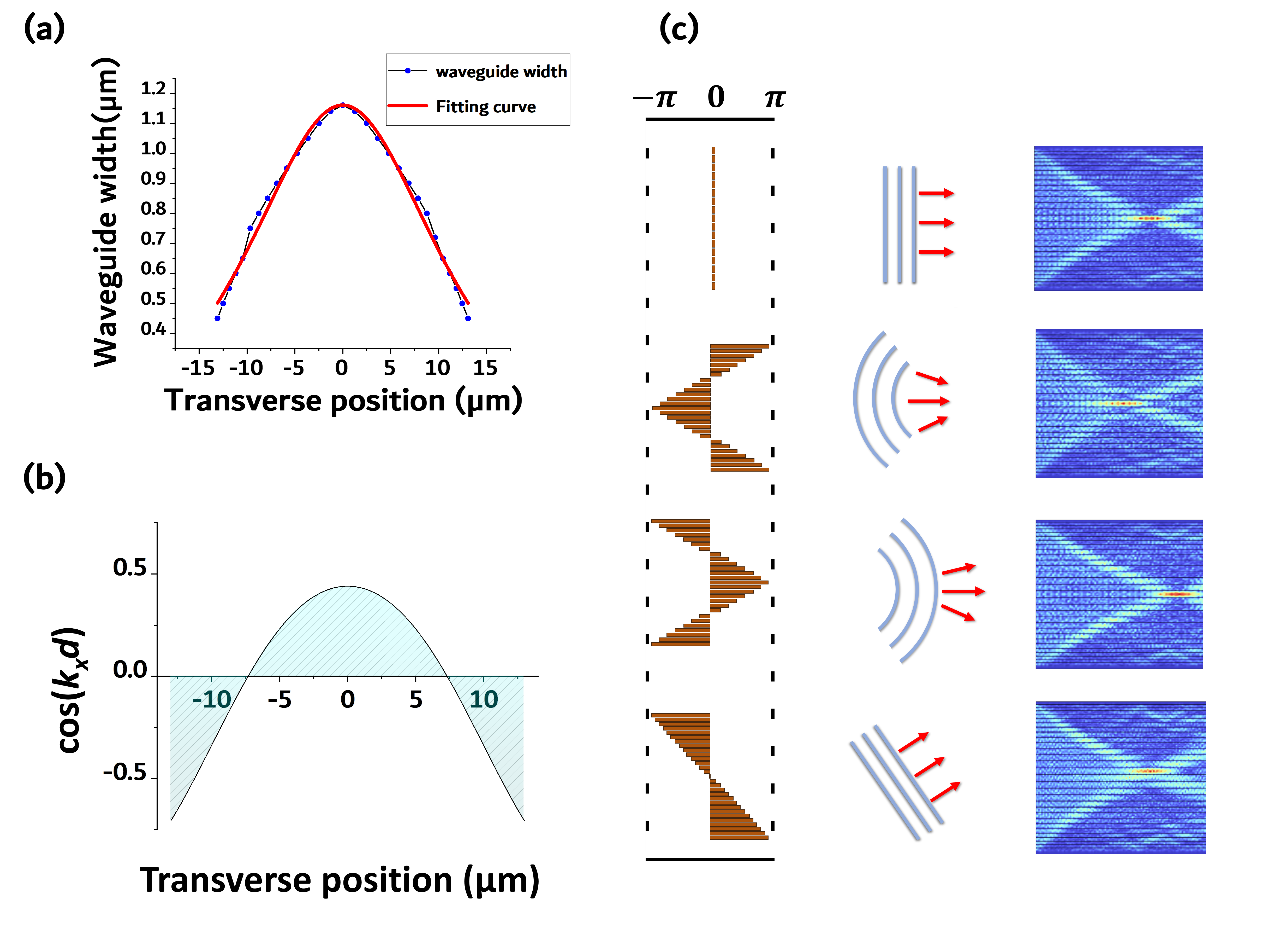


Figure S2: Design and simulation results for spacing-varied SiN waveguide array consists of 29 waveguides, the wavelength and mode considered here is 1550 nm and TM_0_ mode, respectively. (a) Profile of waveguide width along the transverse direction, with a fitting curve that utilizes the hyperbolic secant function. (b) cos(k_x_d) profile of the SiN spacing-varied waveguide arrays. (c) Simulation result of light propagation in spacing-varied waveguide arrays with varying input phase profiles.

**III. Red light transmission in outmost waveguide**

In addition to showing the results with light injection from the center and neighboring ports (Fig. 4c and 4d), we also injected light into other ports during our tests. Let's consider the outermost waveguide as an example. For the TE_1_ mode at 650 nm red light in a spacing-varied SiN waveguide array with an etching depth of 110 nm, the diffraction coefficient in the region near the outermost waveguide approaches zero (Fig. S3(a)). Consequently, the transmission of the light field within that waveguide is expected to exhibit non-diffractive propagation, which was experimentally observed (Fig. S3(b)).


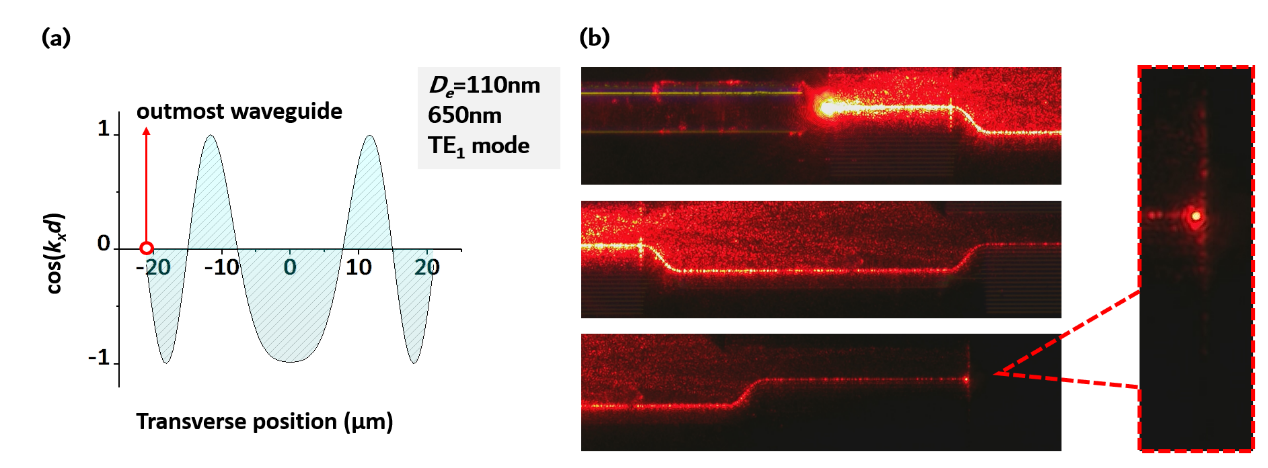


Figure S3: Experiment results for red light propagation in outmost waveguide. (a) cos(k_x_d) profile of the SiN spacing-varied waveguide arrays with an etching depth D_e_=110 nm at 650 nm, the propagation mode was TE_1_. (b) Input facet, surface scattering, and output facet of the SiN spacing-varied waveguide array with 650-nm light input from the outmost waveguide.

**IV. Characterization of the fabricated waveguide arrays**

To assess the quality of the fabricated samples, a simple straight reference waveguide structure was utilized, as depicted in Figure S4(a)-(b). Both the input and output facets of the samples were polished using various levels of fine-grit sandpaper. The SiN film has a thickness of 290 nm, with an etching depth of 110 nm. We measured the loss of the reference straight waveguides at 1550 nm, considering the TM_0_ propagation mode. The length of the straight waveguide is approximately 1.5 mm. The results are presented in Fig. S5, indicating that the insertion loss of the straight waveguide ranges from -15.32 dB to -12.64 dB. Considering the relatively short length of the straight waveguide, the observed loss is mainly attributed to coupling. In this study, we did not employ spot size converters or grating couplers for coupling, as we needed to investigate wavefront propagation with different wavelengths, modes, and polarizations. However, this approach led to significant loss during the coupling process. In future design iterations, we plan to optimize the performance of the coupler to enhance device efficiency for specific wavelengths, modes, and polarizations. Regarding the efficiency of the waveguide arrays, the efficiency of the 360-μm long waveguide array was evaluated. In this arrayed waveguide, 82% of the power was concentrated into the center waveguide. When subjected to an input power of 1 mW, an output power of 16.22 μW was measured in the center waveguide, corresponding to a loss of approximately -17 dB.

In our design, the input waveguides were initially widely separated to avoid crosstalk (Fig. S4(c)). The connection region between the input waveguide region and the spacing-varied waveguide array was designed to reduce crosstalk between adjacent waveguides. The total length along the propagation direction is 80 μm, with each input waveguide having a width of 1 μm. Tapered waveguides with a total length of 10 μm were adopted to achieve the junction between the arrayed waveguide and the 1-μm wide input waveguide, minimizing crosstalk as much as possible. Regarding the crosstalk evaluation of the waveguide array, we conducted measurements on the 360-μm long arrayed waveguide. Specifically, we quantified the crosstalk between the center waveguide and its adjacent waveguide, yielding a value of -11.97 dB.


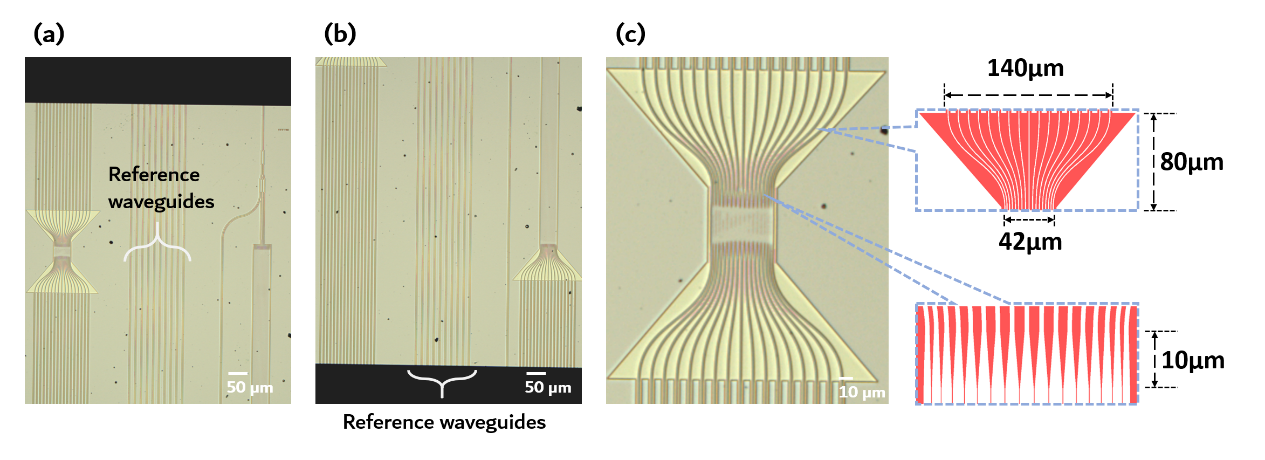


Figure S4: (a) Microscope images of the fabricated samples near the input facet. (b) Microscope images of the fabricated samples near the output facet. (c) Microscope images of the fabricated waveguide array, and illustration of the connection region between input waveguide and arrayed waveguide.


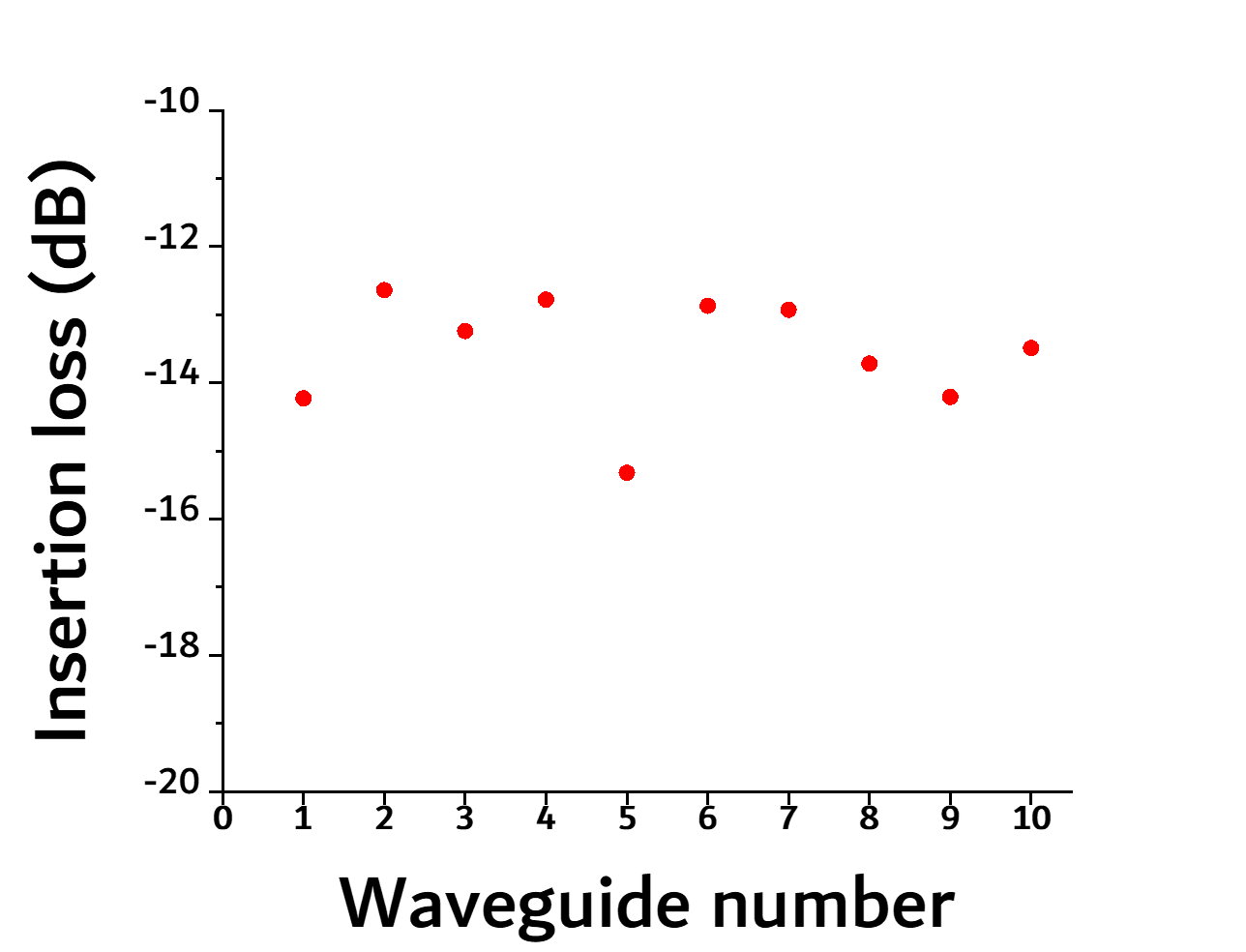


Figure S5: Insertion loss of the straight reference waveguides.
